# Effects of leaf wetness duration and temperature on infection of *Prunus* by *Xanthomonas arboricola* pv. *pruni*

**DOI:** 10.1371/journal.pone.0193813

**Published:** 2018-03-07

**Authors:** Gerard Morales, Concepció Moragrega, Emilio Montesinos, Isidre Llorente

**Affiliations:** Institute of Food and Agricultural Technology-XaRTA-CIDSAV, University of Girona, Girona, Spain; Gyeongnam National University of Science and Technology, REPUBLIC OF KOREA

## Abstract

*Xanthomonas arboricola* pv. *pruni* is the causal agent of bacterial spot disease of stone fruits and almond. The bacterium is distributed throughout the major stone-fruit-producing areas of the World and is considered a quarantine organism in the European Union according to the Council Directive 2000/29/EC, and by the European and Mediterranean Plant Protection Organization. The effect of leaf wetness duration and temperature on infection of *Prunus* by *X*. *arboricola* pv. *pruni* was determined in controlled environment experiments. Potted plants of the peach-almond hybrid GF-677 were inoculated with bacterial suspensions and exposed to combinations of six leaf wetness durations (from 0 to 24 h) and seven fixed temperatures (from 5 to 35°C) during the infection period. Then, plants were transferred to a biosafety greenhouse, removed from bags, and incubated at optimal conditions for disease development. Although leaf wetness was required for infection of *Prunus* by *X*. *arboricola* pv. *pruni*, temperature had a greater effect than leaf wetness duration on disease severity. The combined effect of wetness duration and temperature on disease severity was quantified using a modification of the Weibull equation proposed by Duthie. The reduced-form of Duthie’s model obtained by nonlinear regression analysis fitted well to data (*R* = 0.87 and *R*^*2*^_*adj*_ = 0.85), and all parameters were significantly different from 0. The estimated optimal temperature for infection by *X*. *arboricola* pv. *pruni* was 28.9°C. Wetness periods longer than 10 h at temperatures close to 20°C, or 5 h at temperatures between 25 and 35°C were necessary to cause high disease severity. The predictive capacity of the model was evaluated using an additional set of data obtained from new wetness duration-temperature combinations. In 92% of the events the observed severity agreed with the predicted level of infection risk. The risk chart derived from the reduced form of Duthie’s model can be used to estimate the potential risk for infection of *Prunus* by *X*. *arboricola* pv. *pruni* based on observed or forecasted temperature and wetness duration.

## Introduction

*Xanthomonas arboricola* pv. *pruni* is the causal agent of bacterial spot disease of stone fruits and almond [1,2], which are some of the most economically important tree crops worldwide [3]. The disease was first described in the USA in 1903 [4] and today it is distributed throughout the major stone-fruit-producing areas of the World [5]. Although the bacterium is considered a quarantine organism in the European Union, according to the Council Directive 2000/29/EC, and by the European and Mediterranean Plant Protection Organization (EPPO) [1], the pathogen is currently spreading in many European countries, which have reported local outbreaks [6,7].

*Xanthomonas arboricola* pv. *pruni* affects a wide range of species of *Prunus*, including fruit crops, such as plum, nectarine, peach, apricot, cherry, almond, their hybrids, as well as ornamental species, such as cherry laurel [8,9]. The pathogen mainly causes lesions on leaves and fruits, but twig infections, which can result in perennial cankers in some host species, are sometimes observed in spring or summer. The economic impact of the disease depends on reduced quality and marketability of fruits, reduced orchard productivity, and increased costs of nursery production [10].

Quarantine regulation of *X*. *arboricola* pv. *pruni* by the EU and EPPO is aimed to restrict the introduction and dissemination of this pathogen [1]. Disease control is currently limited to preventive copper spray applications in areas already affected by the disease [8,9,11]. Limitations on copper usage have been defined by the potential for its accumulation in soil, the selection for pathogen resistance [12] and the phytotoxicity in some stone-fruit crops, such as peach and nectarine [13]. In order to minimize the negative effects of copper and to optimize the effectiveness of control measures, disease forecasting models, based on infection risk, can be used to guide the accurate timing of copper applications. Plant disease forecasting models help growers in the decision-making process as an alternative to a fixed spray schedule. Disease forecasters have been successfully implemented in the management of many plant diseases, in which the number of pesticide sprays is reduced in comparison to a fixed spray schedule, but with similar efficacy of disease control [14–16].

Most forecasting models of bacterial plant diseases are based on two processes: i) a temperature-dependent multiplication process of epiphytic populations to provide inoculum; followed by, ii) the occurrence of favorable weather conditions that allow infections [17–20]. A similar approach can be used in the development of a forecasting model of bacterial spot disease of stone fruits. The effect of temperature on the growth of *X*. *arboricola* pv. *pruni* was determined *in vitro* [21,22]. A model for predicting *X*. *arboricola* pv. *pruni* growth as a function of temperature was developed [22], which can be used to estimate the epiphytic inoculum potential of the pathogen. The study revealed that *X*. *arboricola* pv. *pruni* is able to grow within the temperature range from 5 to 35°C and that optimal temperatures for bacterial multiplication are between 25 and 33°C. Regarding the weather conditions conducive to bacterial infections and disease development, different studies agree on the importance of warm temperatures and wetness periods (rainfall, irrigation or dew) for the infection of *Prunus* by *X*. *arboricola* pv. *pruni* [1,9,23,24,25]. The need of wetness for infection is also supported by experiments performed under controlled environment conditions at optimal temperatures for *X*. *arboricola* pv. *pruni* growth (20–30°C) [26,27]. Variability in the range of temperature and wetness period duration conducive to infection and disease development has been detected in studies under field conditions [24,25], probably due to the complex interaction of diverse factors affecting the disease development, such as inoculum populations [28], host susceptibility [7,29] and orchard management practices [9].

Quantification of the effect of environmental factors on the processes of infection and colonization by leaf pathogens is usually carried out in experiments conducted in growth chambers, thus allowing isolation of the effects of specific environmental factors. Therefore, to develop a prediction model of infection of *Prunus* by *X*. *arboricola* pv. *pruni*, the combined effects of temperature and wetness period duration should be quantified under controlled environment conditions for the entire range of temperature at which *X*. *arboricola* pv. *pruni* is able to grow.

Polynomial equations are widely used to quantify the combined effects of wetness and temperature on infection of plants by pathogens for a wide variety of diseases [30], primarily fungal diseases [31,32]. However, various parameters estimated in polynomial equations lack a clear biological significance and some variables may be transformed (e.g. using logarithmic or square root transformations) before fitting the equation. To overcome the weaknesses of polynomial equations, nonlinear models based on modified forms of the Weibull function have been proposed [33], in which all parameters can be interpreted to provide information on the mechanisms of disease response. Weibull based models have been used to quantify temperature and wetness requirements for infection in several diseases [34–37]. A similar approach is proposed in the present work to determine the combined effect of temperature and wetness duration on infection of *Prunus* by *X*. *arboricola* pv. *pruni*.

The objectives of this study were to (i) quantify the effects of temperature and leaf wetness duration on infection of *Prunus* by *X*. *arboricola* pv. *pruni*; (ii) develop a model describing these effects; and (iii) evaluate the capacity of this model to forecast bacterial infection risk.

## Materials and methods

### Plant material

Potted plants of the peach-almond hybrid rootstock GF-677 (*Prunus amygdalus* × *P*. *persica*) obtained by micropropagation (Agromillora Catalana, Subirats, Spain) were selected based on its susceptibility to bacterial spot disease. Plants were grown in 0.5 L pots filled with a commercial peat moss/vermiculite/perlite potting mix (type BVU, Prodeasa, Girona, Spain) in a greenhouse and fertilized once a week with a solution of 200 ppm N-P-K (20-10-20). Twenty-centimeter-high plants with 10 to 15 young expanded leaves were used.

### Inoculum production

*Xanthomonas arboricola* pv. *pruni* strain CFBP 5563 isolated from peach in France obtained from CIRM-CFBP (International Center for Microbial Resources—French Collection for Plant-associated Bacteria, Beaucouzé Cedex, France) was used in this study. Bacteria were stored in stock tubes containing yeast-peptone-glucose broth (YPG) [38] supplemented with glycerol (20% wt/vol) at -70°C. The inoculum was prepared from 24-h-old cultures grown on Luria-Bertrani (LB) [39] plates at 27°C. Bacterial colonies were scraped from the cultures, resuspended in sterile distilled water and adjusted to an optical density of 0.3 at 600 nm, which corresponds to 5 x 10^8^ CFU/ml. A viable count of the inoculum suspension was also determined by spreading 0.1 ml of appropriate 10-fold serial dilutions on yeast-peptone-glucose agar (YPGA) [38] plates and incubation for 72 h at 27°C.

### Pathogen inoculation, incubation and disease assessment

Potted plants of the peach-almond hybrid GF-677 were inoculated by spraying 5 x 10^8^ CFU/ml bacterial suspensions supplemented with diatomaceous earth (1 mg/ml) on plant leaves using an airbrush (model Junior Hobby; Sagola, Vitoria-Gasteiz, Spain) operated at 100 kPa. Adaxial and abaxial leaf surfaces were sprayed until runoff.

Inoculated plants were covered by plastic bags (moist chambers) with the inner side sprayed with distilled water to maintain leaf wetness and transferred immediately to controlled environment chambers (model MLR-350; Sanyo, Gunma, Japan) at constant temperatures of 5, 10, 15, 20, 25, 30 or 35°C in darkness, with a maximum variation of ±1°C for all temperatures. The temperature inside the growth cabinets was monitored and recorded using HOBO Pendant® temperature/light dataloggers (Onset Computer Corp, Pocasset, MA, USA). At 3, 6, 12, 18 or 24 h time intervals, plants were removed from growth chambers and transferred to a biosafety greenhouse maintained at 15 to 25°C with 70 to 80% relative humidity and natural photoperiod for 21 days for disease development. Weather parameters inside the biosafety greenhouse were monitored with a datalogger (CR10X, Campbell Scientific Ltd., UK) connected to combined temperature-relative humidity (model HMP35C) and leaf wetness (model 237) sensors. Plants sprayed with sterile distilled water and incubated at 25°C during 24 h of leaf wetness were used as negative controls. When plants were introduced into the greenhouse, the plastic bags were removed and plants were exposed to a smooth airflow supplied by an electric fan until the leaf surface was dry (30 min). Plants corresponding to 0 h of wetness duration were inoculated directly in the greenhouse and the leaf surface was dried immediately after inoculation.

Disease severity was assessed 21 days after inoculation on the five youngest completely formed leaves at the moment of inoculation. The new leaves formed after the inoculation, during the incubation in the greenhouse, were not considered. A 0-to-5 scale severity index was used, corresponding to a leaf area affected by 0, 1, 3, 6, 12 and ≥ 24%, respectively [24]. Disease severity (*S*) was calculated for each plant according to the formula: $S=\lceil (\sum_{n=1}^{N}I_{n})/N\times 5\rceil \times 100$, where *I*_*n*_ is the severity index for each leaf, *N* is the number of leaves per plant, and *5* is the maximum severity index value in the scale.

A completely randomized experimental design with subsampling was used. The treatment layout was a factorial arrangement with seven temperatures (5, 10, 15, 20, 25, 30 and 35°C) and six wetness periods (0, 3, 6, 12, 18 and 24 h) for a total of thirty six treatments. Each treatment consisted of five plants and five inoculated leaves were evaluated in a plant. The experiment was repeated twice.

### Data analysis and model development

Averaged values of disease severity over the five plants per temperature-wetness combination were standardized using the maximum value observed in each experiment to compare both repetitions. Therefore, the relative disease severity (*S*’) ranged from 0 to 1. The effect of experiment replicate was determined by analysis of variance using the general linear models (GLM) procedure of SPSS v.23 (IBM Corp., Armonk, NY). Previously the homogeneity of variance and normality were tested. As there were no significant differences between the two runs of the experiment for relative disease severity, the following analysis were performed with averaged data over the two runs.

The combined effects of leaf wetness duration (*w*) and temperature during the wet period (*t*) on relative disease severity were evaluated using the nonlinear model proposed by Duthie [33], which is based on a modified form of a Weibull function and it is described by the nonlinear equation of the form:

$$S^{\prime }=f(w,t)=f(t)\times (1-exp\left\{-{\left[B(w-C)\right]}^{D}\right\})$$ (1)

Where *w* is the leaf wetness duration (h) and *t* is the temperature (°C) during the wetness period. The upper asymptote is defined by the expression *f(t*), which characterizes the upper limit on the responses as wetness duration is extended, and it has the following equation:

$$f\left(t\right)=E'\times \left\{\text{exp}\left[\frac{\left(t-F\right)G}{H+1}\right]\right\}/\left\{1+\text{exp}\left[\left(t-F\right)G\right]\right\}$$ (2)

in which

$$E'=E\times [\frac{H+1}{H}]H^{1/(}{}^{H+1)}$$ (3)

*f(t)* characterizes the upper limit of the response when *w* is large. Each parameter in Eqs 1, 2 and 3 has an epidemiological meaning summarized in Table 1. The optimum temperature is given by:

$$t_{opt}=F-\left(1/G\right)\text{ln}\left(H\right)$$ (4)

**Table 1:** Summary of epidemiological significance of parameters in the model proposed by Duthie [33], Eqs 1, 2, and 3, which describes the response of foliar parasites to the combined effects of temperature and duration of wetness.

Parameter	Epidemiological significance
B	Intrinsic rate of increase in response with respect to wetness duration (0 < *B* < 1)
C	Length of delay before the start of the processes that drive the response
D	Period of wetness duration in which the response decelerates (*D* > 0)
E	Maximum response that occurs at the optimum temperature (*E* > 0)
F	Directly proportional to the optimum temperature
G	Intrinsic rate of decline from the maximum as temperature deviates from the optimum
H	Difference in the rate of acceleration in the decline from the maximum as temperature increases or decreases from the optimum

However, the iterative regression procedure failed to converge on a solution when the model included too many parameters. Overparameterization was assessed by the magnitudes of standard errors and correlation coefficients between estimated parameters. The model was simplified by fixing parameter *C* in Eq 1 to a value of 0, assuming that bacteria respond immediately to the increase in leaf wetness duration; and parameter *E* in Eq 3 was fixed to a value of 1, because the disease was measured on a scale of 0 to 1. Moreover, the asymmetry in the temperature response on infection, characterized by parameter *H*, may not be measured when under field conditions at high temperatures wetness is rare [31], and in this case asymmetry may be disregarded. It follows that *H* = 1, *t*_*opt*_ = *F*, *E′* = 2*E*. Eq 1 can be rewritten as [36]:

$$S'=f\left(w,t\right)=\left[1–\text{exp}{\left(–Bxw\right)}^{D}]/\text{cosh}[\left(t–F\right)G/2\right]$$ (5)

Duthie’s model and its simplification, Eqs 1 and 5, were fitted to data. Regressions were based on mean infection data for each combination of temperature and wetness duration rather than on pooled data to reduce data variability and improve curve fitting [40]. Parameters of Duthie’s models, Eqs 1 and 5, were estimated using the sequential quadratic programming method of the nonlinear regression in SPSS v.23 (IBM Corp., Armonk, NY).

From the results obtained, the reduced form of Duthie, Eq 5, was selected for further analysis. Several criteria were used to evaluate this model: (i) randomness and normality of residuals; (ii) goodness of fit between estimated and observed values; and (iii) standard deviation around the regression lines. The nonlinear regression obtained with the Eq 5 was evaluated by performing a linear regression analysis between predicted (*y*) and observed (*x*) values as paired observations. The linear regression was analyzed by the coefficient of determination (*R*^*2*^) and adjusted coefficient of determination (*R*^*2*^_*adj*_), and testing the significance of the difference in the intercept from 0 and the slope from 1.

The capacity of the model for predicting the infection risk was determined using data derived from an additional experiment with a new set of temperature and wetness duration combinations, different from those used for model development. The new set of incubation temperatures were 7.5, 12.5, 17.5, 22.5, 27.5 and 32.5°C for wetness periods of 2, 5, 10, 20 or 24 h. The experiment was performed as described previously, with a total of 24 temperature-wetness duration combinations. Each treatment consisted of five replicates (plants) and five inoculated leaves were evaluated in a plant. The experiment was performed once. The predictions of the model at each temperature-wetness combination were compared with the observed disease severity values using the Pearson’s correlation coefficient and an analysis of frequency was performed for the two categories proposed for model prediction values and for observed disease severity values, respectively.

## Results

No lesions were observed on non-inoculated leaves and few infections occurred on inoculated plants incubated without leaf wetness. The disease severity values corresponding to 0 h of wetness duration (5.5 and 5.6% in experiments 1 and 2, respectively) were subtracted from the disease severity observed for each temperature-wetness combination, as it was probably due to the effect of the residual wetting times during the drying process. The highest disease severity was observed at 30°C for 24 h of wetness in both experiments, although maximum disease severity values were significantly different (76 and 40% in experiments 1 and 2, respectively). Disease severity values were standardized using the maximum severity observed in each experiment to make the repetitions comparable. No differences in relative disease severity were observed between the two independent experiments (*P* = 0.603), allowing the data from two experiments to be pooled for model development.

**Fig 1:**
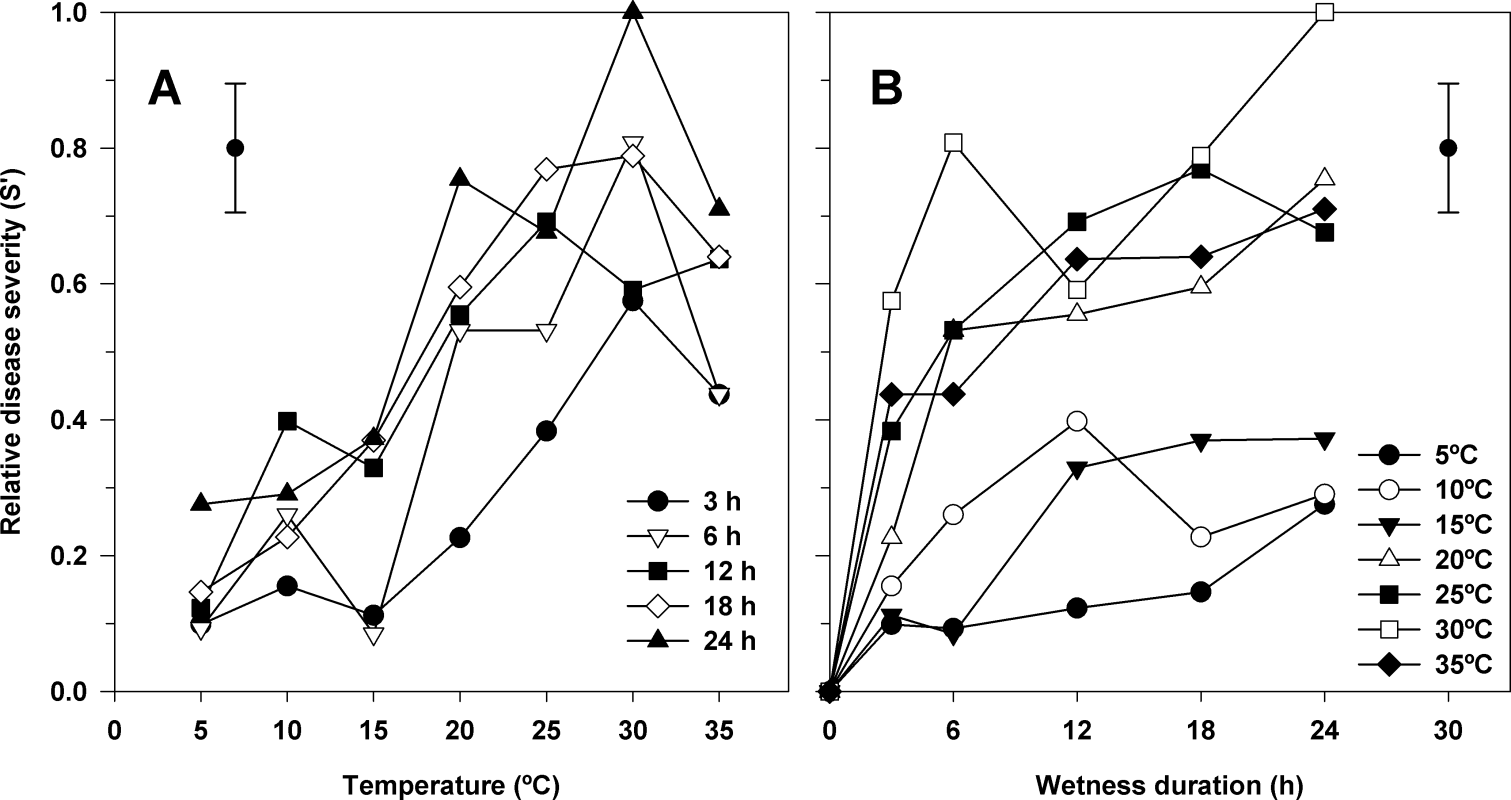
Effect of temperature (A) and leaf wetness duration (B) on relative disease severity caused by *Xanthomonas arboricola* pv. *pruni* on peach-almond hybrid GF-677 plants 21 days after inoculation. Symbols represent the mean relative disease severity of two experiments and five plants per experiment. Error bars in the upper left (A) or right (B) corner represent the mean standard error.

Relative disease severity was affected by temperature during the wetness period. The curves for the different wetness periods evaluated from 5 to 35°C showed similar shapes, although disease severity values differed between them (Fig 1A). The optimal range of temperature for bacterial infection was from 20 to 35°C, with a maximum at 30°C. Below 30°C, an increase of temperature resulted in a gradual increment of disease severity; while above 30°C, there was a slight decrease in the relative disease severity.

Wetness was required for infection of *Prunus* by the pathogen. In general, increasing wetness period duration from 0 to 12 h increased the relative disease severity (Fig 1B). Longer wetness periods, from 12 to 24 h resulted in no or slight increase in relative disease severity. At low temperatures (5, 10, and 15°C) the disease severity increased progressively from 0 without wetness to 0.28, 0.29, and 0.37 under 12 to 24-h-wetness period, respectively. At temperatures from 20 to 35°C, the disease severity also increased with wetness duration increase up to 12–24 h, but high values of disease severity (above 0.4) obtained under short wetness periods (3 or 6 h), which were higher than the maximum disease severity observed at low temperatures under longer wetness periods.

Estimated parameters of the model proposed by Duthie, Eq 1, are presented in Table 2. Parameters *B*, *D*, *F* and *G* were significantly different from zero at *P* < 0.05, whereas parameter *H* presented high standard error and did not differ from zero. Moreover, parameter *H* was highly correlated with parameter *G* and *F* according to Pearson correlation coefficient (*R* = 0.963 and 0.948, respectively). The optimum temperature for the infection of peach-almond hybrid GF-677 plants by *X*. *arboricola* pv. *pruni* calculated with Eq 4 was 30.1°C.

**Table 2:** Estimated parameter values of Duthie’s model, Eq 1, describing the relative disease severity (*S*’) caused by *Xanthomonas arboricola* pv. *pruni* on peach-almond hybrid GF-677 plants based on combined effects of temperature (*t*) and duration of leaf wetness (*w*). In the model $S'=f(w,t)=f\left(t\right)\times (1-\exp\left\{-{\left[B\times w\right]}^{D}\right\})$, where f(t)=E'×{exp[(t-F)G/(H+1)]}/{1+exp[(t-F)G]}, in which $E^{'}=[(H+1)/H]H^{1/(H+1)}$.

Parameter^z^	Estimate	Standard Error	Lower Bound	Upper Bound	*t*-test	*P* > *t*
B	0.167	0.032	0.101	0.234	5.149	< 0.0001
D	0.582	0.111	0.356	0.808	5.265	< 0.0001
F	33.053	2.230	28.499	37.607	14.824	< 0.0001
G	0.266	0.080	0.103	0.429	3.339	0.0023
H	2.514	1.628	-0.811	5.838	1.544	0.1330

^z^ Parameters as defined in the main text, Eq 1.

The reduced four-parameter version of the model, Eq 5, was obtained disregarding parameter *H* (*H =* 1). Estimated values are presented in Table 3, in which all parameters (*B*, *D*, *F* and *G*) were estimated precisely, significantly different from zero at *P* < 0.05, and correlation coefficients between parameters were low (*R* < 0.605). The optimum temperature is represented by parameter *F*, which was 28.9°C. This model, corresponding to Eq 5, was selected as all parameters were significant, the standard errors obtained were lower than those obtained using Eq 1, high correlations between parameters were absent, and the number of parameters were fewer than the model obtained with Eq 1.

**Table 3:** Estimated parameter values of the reduced form of Duthie’s model, Eq 5, describing the relative disease severity (*S*’) caused by *Xanthomonas arboricola* pv. *pruni* on peach-almond hybrid GF-677 plants based on combined effects of temperature (*t*) and duration of leaf wetness (*w*). In the model $S'=f(w,t)=[{1–\exp\times p(–Bxw)}^{D}]/cosh[(t–F)G/2]$.

Parameter^z^	Estimate	Standard Error	Lower Bound	Upper Bound	*t*-test	*P* > *t*
B	0.145	0.023	0.098	0.193	6.272	< 0.0001
D	0.553	0.097	0.354	0.752	5.670	< 0.0001
F	28.919	0.850	27.184	30.653	34.008	< 0.0001
G	0.195	0.018	0.159	0.231	11.116	< 0.0001

^z^ Parameters as defined in the main text, Eq 5.

**Fig 2:**
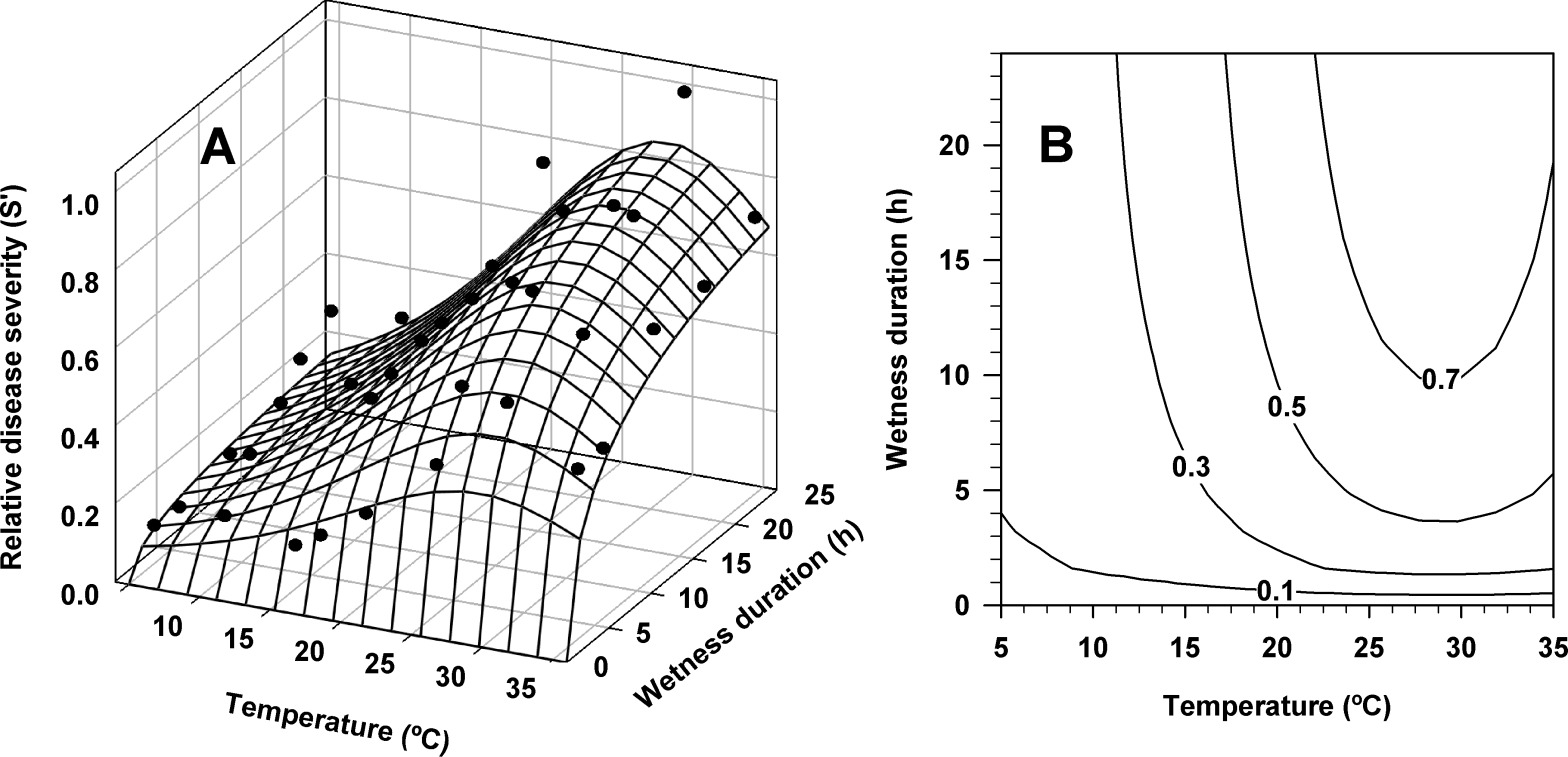
Three-dimensional response (A) and contour plot (B) of the combined effects of temperature and leaf wetness duration on relative disease severity caused by *Xanthomonas arboricola* pv. *pruni* on peach-almond hybrid GF-677 plants. The predicted values were calculated using the reduced form of Duthie’s model, corresponding to Eq 5. (A) The black points correspond to mean relative disease severity of two independent experiments and five plants per experiment observed 21 days after bacterial inoculation. (B) An area represents relative disease severity values lower than or equal to the label on the contour line on the right of the area.

Two- and three-dimensional representations of the response of the reduced form of Duthie’s model, Eq 5, are given in Fig 2. Relative disease severity increased sigmoidally with increasing wetness duration, while the temperature response was unimodal, with the optimal temperature at 28.9°C. The model provided good prediction for all wetness duration-temperature combinations. The Pearson correlation coefficient (*R*) between observed and predicted values was 0.93, and the coefficients of determination *R*^2^ and *R*^*2*^ adjusted for the nonlinear regressions were 0.88 and 0.85, respectively.

**Fig 3:**
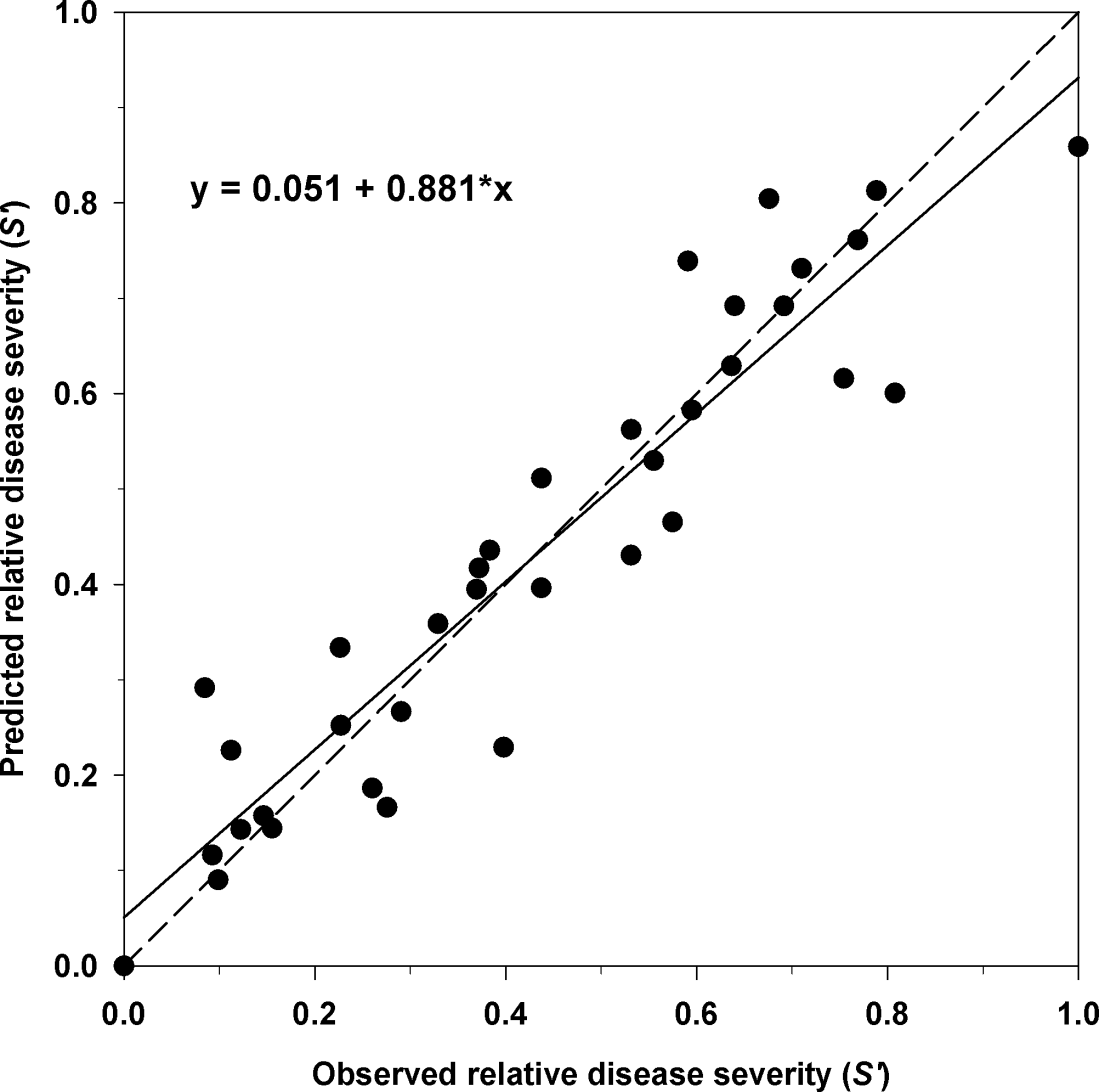
Observed relative disease severity versus estimated values with the reduced form of Duthie’s model, Eq 5. The regression line (solid) is slightly different from the dashed line, which corresponds to a fitted line with an intercept of 0 and a slope of 1.

The linear regression of the predicted values against the observed relative disease severity also showed the good relationship between observed and predicted values (Fig 3). The coefficients of determination *R*^2^ and *R*^*2*^ adjusted for the linear regressions were both 0.88. Although the intercept was not significantly different from 0 (*P* = 0.0823), the slope was slightly different from 1 (*P* = 0.0407). A slight underprediction of the model at high disease levels and a slight overprediction at low values were observed.

**Fig 4:**
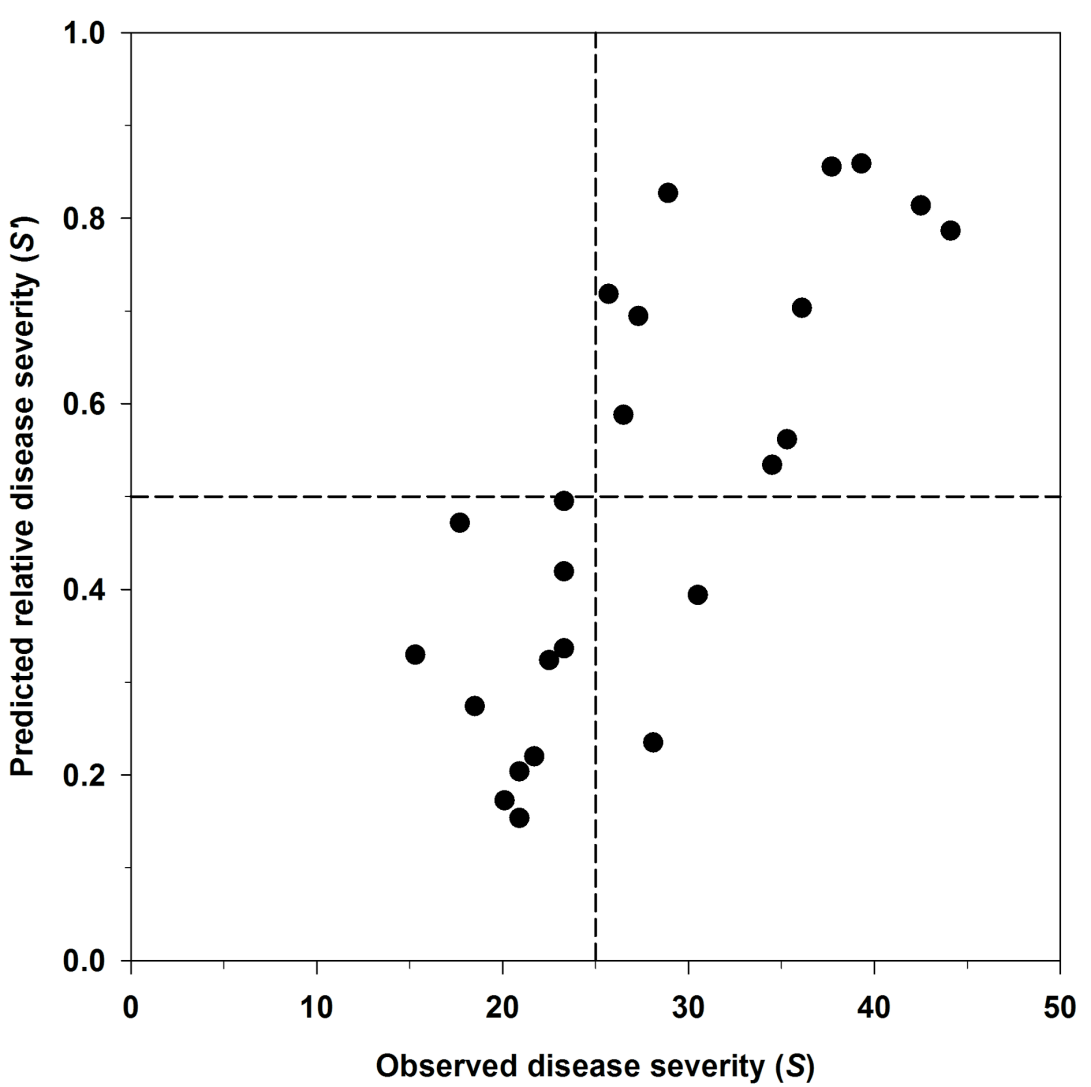
Evaluation of the prediction capacity of the infection model for *Xanthomonas arboricola* pv. *pruni* describing the combined effects of leaf wetness and temperature under greenhouse conditions. Comparison between observed disease severity (*S*, 0–100 range) versus predicted relative disease severity (*S*’, 0–1 range) by the reduced form of Duthie’s model, Eq 5, on GF-677 peach-almond hybrid plants 21 days after inoculation.

The predictive capacity of the infection model was evaluated in a new experiment using an independent set of data with different temperature-wetness duration combinations. Observed disease severity (*S*), based on the leaf area affected by disease, was compared with model predictions for relative disease severity (*S*’), which corresponded to the infection risk index (Fig 4). A positive and high correlation resulted between these two variables (*R* = 0.761). The relationship between predicted relative disease severity (*S*’) and observed severity (*S*) was also used to define a risk threshold for infection. Disease severity up to 25% was considered low since it corresponded, on average, to plants with less than 2% leaf area infected. There was a significant relationship between observed disease severity lower than 25% and predicted relative severity lower than 0.5; and also between observed disease severity higher than 25% and predicted relative severities from 0.5 to 1. Consequently, the value 0.5 of relative severity predicted by the model (*S’*) was proposed as the infection risk threshold. Accordingly, two levels of infection risk were established: low (*S’* < 0.5) and high (*S’* ≥ 0.5). On the basis of the infection risk levels, model predictions agreed on 22 of 24 cases (92%), 11 for low and 11 for high risk predictions. Only 2 mismatches were observed, corresponding to temperature-wetness combinations predicted as low infection risk that expressed medium severity values.

## Discussion

The combined effects of leaf wetness duration and temperature on infection of *Prunus* by *X*. *arboricola* pv. *pruni* were determined under controlled environment conditions for the entire range of temperatures at which the bacterium is able to grow [21,22]. Optimal temperatures for infection by *X*. *arboricola* pv. *pruni* conducive to severe bacterial spot disease (*S’* > 0.5) ranged from 20 to 35°C, with a maximum at 30°C. Low temperatures (10–15°C) resulted in low relative disease severity (*S’* < 0.5). Plants incubated at 5°C developed few lesions which could be related to the effect of the residual free water on the leaf surface and the water congestion in leaf tissues during the drying process in the greenhouse. Optimal temperatures for infection on *Prunus* agreed with optimal temperatures for *X*. *arboricola* pv. *pruni* growth, with maximum bacterial growth rates at temperatures from 20 to 30°C [21,22].

Leaf wetness durations from 3 to 6 hour were sufficient for infection and cause high disease severity at optimal temperatures (20–35°C). Longer wetting periods resulted in increased disease severity, and wetness periods from 12 to 24 h had the maximum effect on infection by the bacterium. The minimum leaf wetness period required for infection by *X*. *arboricola* pv. *pruni* depended on temperature, being short (3 h) at optimal temperatures and longer (6 to 12 h) at temperatures below 20°C. These results confirm that wetness is required for the infection of *X*. *arboricola* pv. *pruni* on *Prunus* [25–27]. At 0 h wetness duration no disease symptoms were observed on the majority of plants. The few infections that did occur with 0 h wetness can likely be explained by residual water from the inoculation of the pathogen suspension, which remained in stomatal tissues or on the leaf surface during the drying process in the greenhouse. Some disease symptoms were also observed on plants dried immediately after inoculation in previous studies [26]. Similarly, only 5 min of leaf wetness were sufficient for *X*. *arboricola* pv. *juglandis* to infect water-congested young walnut fruits [41].

The results obtained in this study under controlled environment conditions are consistent with field observations, which concluded that warm temperatures accompanied by frequent rains or heavy dews play an important role in disease development [1, 9, 24]. In fact, temperature, rainfall and wetness are considered key factors for infection by *X*. *arboricola* pv. *pruni* in peach orchards [24,25,42].

The combined effects of temperature and wetness duration on infection by *X*. *arboricola* pv. *pruni* were evaluated and quantified using nonlinear equations proposed by Duthie [33], which are a variation of the Weibull equation. Nonlinear models proposed by Duthie [33] provide a more parsimonious description of empirical data than models that comprise polynomial equations and all parameters have an epidemiological significance. Many infection models use regression equations based on polynomials [31,43], but model parameters have no biological meaning and thus, poorly describe the response of a biological process [33]. Moreover, polynomial models are only reliable in the range of temperatures and wetness used to construct them; otherwise, unrealistic results are obtained if the equations are applied outside this range [36]. The model obtained in this work accurately described the relationship between wetness duration and temperature on disease severity, according to predicted and observed comparisons for disease severity and levels of infection risk. The model had a unimodal response, but some simplifications were performed to avoid overparameterization in the nonlinear regression. The parameter *C* (period before the start of the response) was fixed to 0 assuming that bacteria respond immediately to the increase in leaf wetness duration, and parameter *E* (upper limit of the response) was fixed to 1, because the disease was measured as a proportion (0–1). All parameters in the equation were significant, except parameter *H*, which represents the degree of asymmetry in the temperature response. *X*. *arboricola* pv. *pruni* is able to grow *in vitro* at temperatures from 5 to 34°C, but not above 35°C [21,22]. The optimal temperature for its multiplication *in vitro* was 31°C [21,22], which means that the growth curve is skewed negatively. If the response of *Prunus* infection by *X*. *arboricola* pv. *pruni* to temperature were similar to the growth response, the parameter *H* might be >1. However, only one temperature (35°C) was tested above the optimal temperature for infection; therefore, it was difficult to determine the grade of asymmetry of the curves. At least one more temperature above 35°C should be tested (e.g., 40°C) to assure the asymmetry of the response, but leaf wetness and temperatures above 35°C rarely occur under field conditions in temperate regions where *Prunus* species are grown. Consequently, the regression was performed disregarding the asymmetry of the curve (parameter *H* was fixed to 1) [33].

The output of the model developed under controlled environment conditions is based on a quantitative relationship between leaf wetness duration and the temperature during wetness periods on infection by *X*. *arboricola* pv. *pruni*. As a result, the periods of risk for infection can be predicted. The evaluation of the accuracy of the model to determine the levels of disease risk (*S’*), showed that *S’* = 0.5 (in a 0–1 range) could be used as a threshold, above which medium or high disease severity was reached. Days with risk of infection by *X*. *arboricola* pv. *pruni* could, therefore, be identified based on weather parameters. *Xanthomonas arboricola* pv. *pruni* infections can occur with low wetness periods (3 to 6 h) at optimal temperatures, but according to the infection model, at least 10 h wetness at temperatures close to 20°C, or 5 h of wetness at 25°C or higher are necessary to reach the infection risk threshold of *S’* = 0.5. These weather conditions are not frequent in temperate regions where species of *Prunus* are grown. Therefore, days with favorable weather conditions to trigger bacterial spot infections may be related to rainfall events and/or long wetting periods in spring and summer. Epidemiological studies performed in Italian peach orchards [24,42] support our results. Primary infections by *X*. *arboricola* pv. *pruni* were observed in peach orchards when at least 3 successive rainy days occurred, with a mean temperature between 14 and 19°C, and the progress of disease severity on leaves was closely correlated with the number of rainy days after disease onset [24]. Consequently, wetness periods over 24 h could be considered by the additive effect of daily infection risk index, obtaining a cumulative infection risk index. The use of cumulative risk indices in plant disease-warning systems gives more accurate predictions and helps to explain field disease epidemics [30,44].

The model evaluation was performed under controlled greenhouse conditions, but field evaluation and validation are needed before the model can be used as part of a decision support system (DSS) in the management of the bacterial spot disease of stone fruits. The proposed model should be evaluated and validated in areas where the disease is present. The model was developed on the hybrid rootstock GF677 (*Prunus amygdalus* x *P*. *persica*), susceptible to the bacterial spot disease of stone fruits in nurseries, as representative of *s*pecies of *Prunus* which are hosts of *X*. *arboricola* pv. *pruni*. However, field validation trials should be performed on grafted plants of different species of *Prunus*. Variability in cultivar susceptibility within each host species [7,29] could also be taken into account in the forecasting model.

The model presented in this manuscript determines the suitability of weather conditions for the infection of *Prunus* by *X*. *arboricola* pv. *pruni*. Upon the evaluation and validation of the model under field conditions, it could be included as the second component of a bacterial spot of stone fruit disease forecasting model, in a similar approach as Maryblight [19], Cougarblight [18], and Billing’s integrated system [20] forecasting models for fire blight of apple and pear caused by *Erwinia amylovora*. The first component of the forecasting model, corresponding to the inoculum potential, is based on the model for predicting *Xanthomonas arboricola* pv. *pruni* growth as a function of temperature [22], which can be used to predict the epiphytic potential inoculum. The integration of both components is fundamental to accurately predict bacterial infections, since both inoculum and favorable environmental conditions are required to come together at the same time for successful infection occurrences.

The forecasting model developed in this study could be used in warning systems for a rational timing of copper sprays for bacterial spot disease control, but also for early detection of disease outbreaks in quarantine and surveillance strategies. Disease risk maps elaborated from model predictions could help to define areas with risk of infection by *X*. *arboricola* pv. *pruni*.

## Conclusions

The work presented here quantifies the effects of leaf wetness duration and temperature on infections of *Prunus* by *X*. *arboricola* pv. *pruni*. Leaf wetness was required for infection of *Prunus* by *X*. *arboricola* pv. *pruni*. However, temperature had a greater effect than leaf wetness duration on the disease severity. Disease severity increased with increasing in temperature until the optimal at 28.9°C, and with increasing leaf wetness duration up to 12 h. The combined effects of leaf wetness duration and temperature on disease severity have been described using the reduced-form of Duthie’s model. An infection risk threshold of *S’* = 0.5 was established for the model. Accordingly wetness periods longer than 10 h at temperatures close to 20°C, or 5 h at temperatures between 25 and 35°C were necessary to cause high disease severity. The obtained model should be evaluated and validated under field conditions to be used as a forecasting model of the potential risk for infections of *Prunus* by *X*. *arboricola* pv. *pruni*.

## References

[1] EPPO/CABI. *Xanthomonas arboricola* pv. *pruni* In: Smith IM, McNamara DG, Scott PR, Holderness M editors. Quarantine Pests for Europe. 2nd ed CAB International, Wallingford, UK; 1997 pp. 1096–1100.

[2] Palacio-Bielsa A, Roselló M, Cambra MA, López MM. First report on almond in Europe of bacterial spot disease of stone fruits caused by *Xanthomonas arboricola* pv. *pruni*. Plant Dis. 2010; 94:786. 10.1094/PDIS-94-6-0786B 30754327

[3] Lamichhane JR. *Xanthomonas arboricola* diseases of stone fruit, almond, and walnut trees: progress toward understanding and management. Plant Dis. 2014; 98:1600–1610. 10.1094/PDIS-08-14-0831-FE 30703892

[4] Smith EF. Observation on a hitherto unreported bacterial disease, the cause of which enters the plant through ordinary stomata. Science. 1903; 17:456–457.

[5] EPPO. Xanthomonas arboricola pv. pruni (XANTPR). EPPO Global Database. 2017. Available from: https://gd.eppo.int

[6] EPPO Diagnostics *Xanthomonas arboricola* pv. *pruni*. EPPO Bulletin. 2006; 36:129–133

[7] Scortichini M. Epidemiology and predisposing factors of some major bacterial diseases of stone and nut fruit trees species. J. Plant Pathol. 2010; 92:S1.73–S1.78.

[8] EFSA Panel on Plant Health. Scientific opinion on pest categorisation of *Xanthomonas arboricola* pv. *pruni* (Smith, 1903). EFSA Journal. 2014; 12:3857

[9] Stefani E. Economic significance and control of bacterial spot/canker of stone fruits caused by *Xanthomonas arboricola* pv. *pruni*. J. Plant Pathol. 2010; 92:99–104.

[10] Janse JD. Bacterial diseases that may or do emerge, with (possible) economic damage for Europe and the Mediterranean basin: Notes on epidemiology, risks, prevention and management on first occurrence. J. Plant Pathol. 2012; 94:S4.5–S4.29.

[11] Garcin A, Rouzet J, Notteghem J-L. *Xanthomonas* des arbres fruitiers à noyau. Edition CTFL; 2005.

[12] Vanneste J, McLaren G, Yu J. Copper and streptomycin resistance in bacterial strains isolated from stone fruit orchards in New Zealand. N. Z. Plant Prot. 2005; 58:101–105.

[13] Lalancette N, McFarland K. Phytotoxicity of copper-based bactericides to peach and nectarine. Plant Dis. 2007; 91:1122–1130. 10.1094/PDIS-91-9-1122 30780652

[14] Madden LV, Ellis MA, Lalancette N, Hughes G, Wilson LL. Evaluation of a disease warning system for downy mildew of grapes. Plant Dis. 2000; 84:549–554. 10.1094/PDIS.2000.84.5.549 30841347

[15] Llorente I, Vilardell P, Bugiani R, Gherardi I, Montesinos E. Evaluation of BSPcast disease warning system in reduced fungicide use programs for management of brown spot of pear. Plant Dis. 2000; 84:631–637. 10.1094/PDIS.2000.84.6.631 30841102

[16] Thomson SV, Schroth MN, Moller WJ, Reil WO, Beutel JA, Davis CS. Pesticide applications can be reduced by forecasting the occurrence of fireblight bacteria. Calif Agr. 1977; 31:12–14.

[17] Beresford RM, Tyson JL, Henshall WR. development and validation of an infection risk model for bacterial canker of kiwifruit, using a multiplication and dispersal concept for forecasting bacterial diseases. Phytopathology. 2017; 107:184–191. doi: 10.1094/PHYTO-04-16-0166-R 27749150 10.1094/PHYTO-04-16-0166-R

[18] Smith T. A predictive model for forecasting fire blight of pear and apple in Washington state. Acta Hortic. 1993; 338:153–160.

[19] Lightner GW, Steiner PW. Maryblyt^TM^: A computer model for predicting of fire blight disease in apples and pears. Comput. Electron. Agric. 1992; 7:249–260.

[20] Billing E. Fire blight risk assessment: Billing’s integrated system (BIS) and its evaluation. Acta Hortic. 1999; 399–406.

[21] Young J, Luketina R, Marshall A. The effects on temperature on growth in vitro of *Pseudomonas syringae* and *Xanthomonas pruni*. J. Appl. Bacteriol. 1977; 42:345–354. 885818 10.1111/j.1365-2672.1977.tb00702.x

[22] Morales G, Llorente I, Montesinos E, Moragrega C. A model for predicting *Xanthomonas arboricola* pv. *pruni* growth as a function of temperature. PLoS One. 2017; 12(5): e0177583 doi: 10.1371/journal.pone.0177583 28493954 10.1371/journal.pone.0177583 PMC5426779

[23] Linvill DE. Use the proper environmental temperature to describe disease. Acta Hortic. 2002; 592:689–694.

[24] Battilani P, Rossi V, Saccardi A. Development of *Xanthomonas arboricola* pv. *pruni* epidemics on peaches. J. Plant Pathol. 1999; 81:161–171.

[25] Garcin A, Vibert J, Leclerc A. *Xanthomonas* sur pêcher. étude des conditions d’infection. Développement de l’outil (1re partie). Infos CTIFL. 2011; 268:26–39.

[26] Zehr EI, Shepard DP, Bridges WC Jr. Bacterial spot of peach as influenced by water congestion, leaf wetness duration, and temperature. Plant Dis. 1996; 80:339–341.

[27] Morales G, Llorente I, Montesinos E, Moragrega C. Basis for a predictive model of *Xanthomonas arboricola* pv. *pruni* growth and infections in host plants. Acta Hortic. 2016; 1–8.

[28] Randhawa PS, Civerolo E. A detached-leaf bioassay for *Xanthomonas campestris* pv. *pruni*. Phytopathology. 1985; 75:1060–1063.

[29] Garcin A, Bresson J. Sensibilité des arbres à noyau au *Xanthomonas*—Bilan de 8 ans d’expérimentation. Infos CTIFL. 2009; 254:30–35.

[30] Magarey RD, Sutton TB. How to create and deploy infection models for plant pathogens In: Ciancio A, Mukerji KG, editors. General concepts in integrated pest and disease management. Dordrecht: Springer Netherlands; 2007 pp. 3–25.

[31] Montesinos E, Moragrega C, Llorente I, Vilardell P, Bonaterra A, Ponti I, et al Development and evaluation of an infection model for *Stemphylium vesicarium* on pear based on temperature and wetness duration. Phytopathology. 1995; 85:586–592.

[32] Gross MK, Santini JB, Tikhonova I, Latin R. The influence of temperature and leaf wetness duration on infection of perennial ryegrass by *Rhizoctonia solani*. Plant Dis. 1998; 82:1012–1016. 10.1094/PDIS.1998.82.9.1012 30856827

[33] Duthie JA. Models of the response of foliar parasites to the combined effects of temperature and duration of wetness. Phytopathol. 1997; 87:1088–1095. 10.1094/PHYTO.1997.87.11.1088 18945004

[34] Carisse O, Bourgeois G, Duthie JA. Influence of temperature and leaf wetness duration on infection of strawberry leaves by *Mycosphaerella fragariae*. Phytopathology. 2000; 90:1120–1125. doi: 10.1094/PHYTO.2000.90.10.1120 18944475 10.1094/PHYTO.2000.90.10.1120

[35] Wu L, Damicone JP, Duthie JA, Melouk HA. Effects of temperature and wetness duration on infection of peanut cultivars by *Cercospora arachidicola*. Phytopathology. 1999; 89:653–659. doi: 10.1094/PHYTO.1999.89.8.653 18944677 10.1094/PHYTO.1999.89.8.653

[36] Arauz LF, Neufeld KN, Lloyd AL, Ojiambo PS. Quantitative models for germination and infection of *Pseudoperonospora cubensis* in response to temperature and duration of leaf wetness. Phytopathol. 2010; 100:959–67. 10.1094/PHYTO-100-9-0959 20701494

[37] Furuya H, Takanashi H, Fuji S, Nagai Y, Naito H. Modeling infection of spring onion by *Puccinia allii* in response to temperature and leaf wetness. Phytopathology. 2009; 99:951–956. doi: 10.1094/PHYTO-99-8-0951 19594314 10.1094/PHYTO-99-8-0951

[38] Boudon S, Manceau C, Nottéghem J-L. Structure and origin of *Xanthomonas arboricola* pv. *pruni* populations causing bacterial spot of stone fruit trees in western Europe. Phytopathology. 2005; 95:1081–1088. doi: 10.1094/PHYTO-95-1081 18943306 10.1094/PHYTO-95-1081

[39] Maniatis T, Fritsch EF, Sambrook J. Molecular cloning: a laboratory manual. 2nd ed Cold Spring Harbor Laboratory, Cold Spring Harbor, NY; 1982.

[40] Sant’Ana AS, Franco BD, Schaffner DW. Modeling the growth rate and lag time of different strains of *Salmonella enterica* and *Listeria monocytogenes* in ready-to-eat lettuce. Food Microbiol. 2012; 30:267–273. doi: 10.1016/j.fm.2011.11.003 22265311 10.1016/j.fm.2011.11.003

[41] Adaskaveg JE, Förster H, Thompson D, Driever G, Connell J, Buchner R. Epidemiology and management of walnut blight. California Walnut Board, Walnut Research Report. 2005; 281–295.

[42] Bugiani R, Giosuè S, Gianni C, Rossi V. Prediction of *Xanthomonas arboricola* pv. *pruni* infection on peaches. IOBC/WPRS Bulletin. 2008; 54:565–569.

[43] Uddin W, Serlemitsos K, Viji G. A temperature and leaf wetness duration-based model for prediction of gray leaf spot of perennial ryegrass turf. Phytopathology. 2003; 93:336–343. doi: 10.1094/PHYTO.2003.93.3.336 18944344 10.1094/PHYTO.2003.93.3.336

[44] Kim KS, Gleason ML, Taylor SE. Forecasting site-specific leaf wetness duration for input to disease-warning systems. Plant Dis. 2006; 90:650–656. 10.1094/PD-90-0650 30781143

